# Hedgehog Signalling Contributes to Trauma-Induced Tendon Heterotopic Ossification and Regulates Osteogenesis through Antioxidant Pathway in Tendon-Derived Stem Cells

**DOI:** 10.3390/antiox11112265

**Published:** 2022-11-16

**Authors:** Guanzhi Li, Ye Deng, Kaiqun Li, Yuchen Liu, Ling Wang, Zhiyong Wu, Chao Chen, Kairui Zhang, Bin Yu

**Affiliations:** 1 Division of Orthopaedics and Traumatology, Department of Orthopaedics, Nanfang Hospital, Southern Medical University, Guangzhou 510515, China; 2 Department of Orthopaedics, Chengdu Second People’s Hospital, Chengdu 610017, China; 3 Biomaterials Research Center, School of Biomedical Engineering, Southern Medical University, Guangzhou 510515, China; 4 College of Traditional Chinese Medicine, Southern Medical University, Guangzhou 510515, China; 5 Department of Traditional Chinese Medicine, Nanfang Hospital, Southern Medical University, Guangzhou 510515, China; 6 Department of Orthopaedics, School of Traditional Chinese Medicine, Southern Medical University, Guangzhou 510515, China; 7 Guangdong Provincial Key Laboratory of Bone and Cartilage Regenerative Medicine, Nanfang Hospital, Southern Medical University, Guangzhou 510515, China

**Keywords:** hedgehog signalling, tendon, heterotopic ossification, antioxidant, reactive oxygen species, tendon-derived stem cells

## Abstract

Heterotopic ossification (HO) is defined as the generation of pathological ectopic bony structures in soft tissues, but the molecular mechanisms of tendon HO are not fully revealed. Hedgehog (Hh) signalling is reportedly critical in hereditary HO. Our study focuses on the role of Hh signalling in the formation of trauma-induced tendon ossification. In this study, samples of healthy tendons and injured tendons from C57BL/6J female mice at 1, 4, 7, and 10 weeks after Achilles tenotomy were collected for quantitative real-time polymerase chain reaction (qRT–PCR) and immunohistochemical analysis (IHC). At 1, 4, 7, and 10 weeks postinjury, tendon samples from the mice administered with vehicle, GANT58 (a GLI antagonist), or SAG (a smoothened agonist) were harvested for micro-CT, histological staining, qRT–PCR, and IHC. Rat tendon-derived stem cells (TDSCs) treated with vehicle, GANT58, or SAG were used to induce osteogenic and chondrogenic differentiation in vitro for qRT–PCR, alkaline phosphatase staining, Alcian blue staining, and reactive oxygen species (ROS) levels measurement. We found that Hh signalling is remarkably activated during the formation of trauma-induced tendon ossification in the model of Achilles tenotomy. The in vitro and in vivo assays both confirm that downregulation of Hh signalling significantly suppresses osteogenesis and chondrogenesis to inhibit tendon ossification, while upregulation of Hh signalling promotes this process. Under osteogenic induction, Hh signalling regulates antioxidant pathway and affects ROS generation of TDSCs. Collectively, Hh signalling contributes to trauma-induced tendon ossification and affects ROS generation through antioxidant pathway in osteogenic differentiation of TDSCs, indicating that targeting Hh signalling by GANT58 may be a potential treatment for trauma-induced tendon ossification.

## 1. Introduction

Tendon ossification is a common manifestation of chronic functional and structural disorders caused by tendon injury. In trauma-related tendon ossification, bony ectopicity frequently causes tendon rupture, restricted mobility, chronic pains, impaired tendon function, and other clinical symptoms [1]. Tendon ossification is a type of heterotopic ossification (HO) that involves the generation of ectopic mineralized bone in soft tissues [2]. HO is pivotal in abnormal tendon conditions (e.g., degenerative changes after mechanical loading, tendon rupture, or trauma surgery) and unusual genetic diseases (e.g., fibrodysplasia ossificans progressiva (FOP), progressive osseous heteroplasia (POH)) [3].

Zhang et al. demonstrate that trauma-induced tendon HO deteriorates the function and biomechanical characteristics of ruptured tendons [4]. The occurrence of tendon ossification can only be mitigated, but not eliminated, by conservative treatment, such as physical therapy and drugs. The current treatment for HO relies heavily on surgical removal of ectopic bony tissues, which inevitably causes additional trauma and thereby easily leads to recurrent HO [5]. In the face of such intractable clinical problems, investigators have intensively explored relevant molecular mechanisms of tendon ossification but have not established a convincing theory to fully elucidate the pathogenesis.

HO can be caused by endochondral ossification [6]. All typical cases of HO concern the development of fibroproliferative lesions involving cells that form HO via the classic endochondral ossification route. Local progenitor/stem cells may be an important cellular pathogenesis of HO [7]. When stimulated, resting osteoprogenitor/stem cells in attacked soft tissues can differentiate into osteoblasts and induce osteoid generation, finally leading to HO [8]. Unfortunately, the cellular pathogenesis of HO occurrence via endochondral ossification is poorly known. Tendon-derived stem cells (TDSCs), one precursor of tendon cells, exhibit self-renewing and multi-differentiating potential in tenogenic, osteogenic, and chondrogenic differentiation [9,10,11]. As reported, osteogenic differentiation of TDSCs is pivotal in tendon HO [12,13]. Even so, the mechanism of erroneous differentiation of TDSCs in the occurrence of HO is not fully clear [14].

The ligands of the hedgehog (Hh) signalling pathway are Hh proteins, activation of which requires a series of processes. Two Hh ligands viz. Indian hedgehog (IHH) and Sonic hedgehog (SHH) can modulate musculoskeletal system development [15]. Hh signalling is initiated by the binding of SHH and IHH to the membrane-binding receptor Patch1 (PTCH1), and thereby its starting of the transmembrane protein Smoothened (SMO) is reversed and, subsequently, the activity of GLI family member transcription factors is regulated, transmitting the signal into the nucleus to activate the transcription of downstream target genes [16]. The recombinant GLI family zinc finger protein 1 (GLI1) is often used as a marker for Hh signal activation [17]. Hh signalling is critical in cartilage and bone growth, and its dysregulation results in bone tumours, osteoarthritis, HO, and other bone diseases [18]. Despite these findings, it has yet to be therapeutically harnessed for musculoskeletal disorders. While Hh signalling is highly correlated with HO, how Hh signalling impacts the maintenance and function of TDSCs in HO has not been reported. Because HO implicates both osteogenic and chondrogenic differentiation [19], the effects of Hh signalling on such differentiation of TDSCs may be critically involved in the physiology and pathobiology of tendon HO.

Reactive oxygen species (ROS) play a regulatory role in cell growth, differentiation, and signalling transduction [20]. However, excessive ROS in the body can cause oxidative stress and lead to various diseases [21]. It has been reported that high levels of ROS affect the differentiation of tendon stem cells and participate in the pathogenesis of tendon HO [22]. Hh signalling can be involved in the mechanism of neurological diseases by acting on ROS [23]. Whether Hh signalling can regulate ROS level and play an antioxidant role in TDSCs during the pathogenesis of tendon HO has not been confirmed.

Herein, with the Achilles tenotomy model, we sought to determine whether Hh signalling is regulated in the progression of trauma-associated nonhereditary HO in tendons and whether positive and negative regulation of Hh signalling affects the formation of trauma-induced tendon HO in vivo. Because Hh signalling is closely connected to the biology of both stem cells and chondrocytes [24], we used TDSCs to induce osteogenic and chondrogenic differentiation with positive and negative regulation of Hh signalling, respectively, investigated the regulatory effect of Hh signalling on the osteogenesis and chondrogenesis of TDSCs and the antioxidant effect of Hh signalling on the osteogenesis of TDSCs.

In summary, we aimed to clarify the impact of Hh signalling on the pathogenesis of trauma-induced tendon HO and whether it can be a new therapeutic target for trauma-induced tendon HO.

## 2. Materials and Methods

### 2.1. Animal Model and Grouping

Female C57BL/6J mice, aged 6–8 weeks, were used to build the Achilles tenotomy model. Firstly, a full transverse cutting was created at the midpoint of the right Achilles tendon in each mouse. Then, we sutured the skin and ensured that the ruptured tendon was inside the skin for self-healing. For sham surgery, a skin incision was made without Achilles tendon injury.

**Figure 1:**
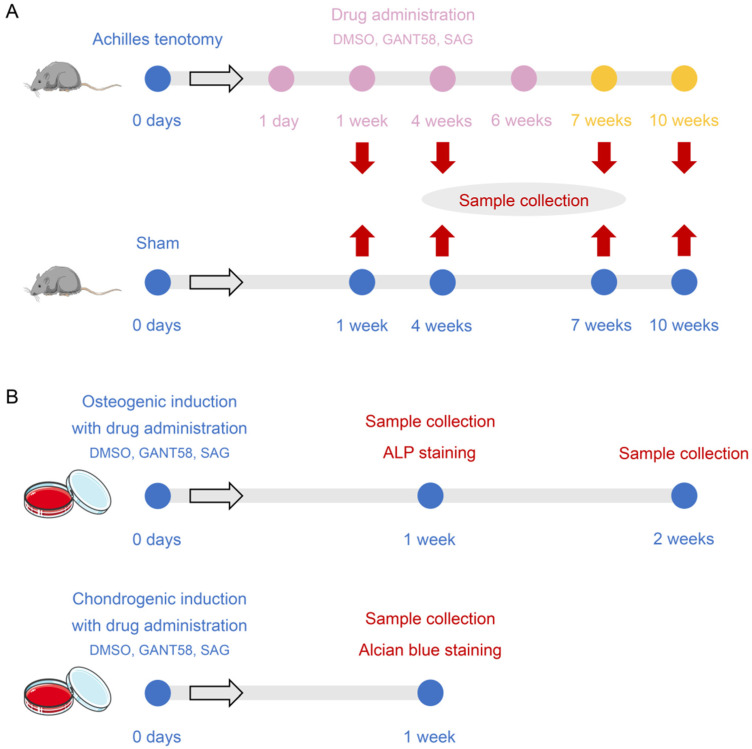
Schematic diagram of experiment design. (**A**) Animal experiment design. (**B**) Cell experiment design.

A total of 236 mice were randomly divided into 16 groups at the same time: 4 vehicle groups euthanized at 1, 4, 7, and 10 weeks after surgery separately (Achilles tenotomy model receiving DMSO administration, *n* = 12 for 1, 4, 7 weeks, *n* = 24 for 10 weeks), 4 GANT58 (a GLI antagonist) groups euthanized at 1, 4, 7, and 10 weeks separately (Achilles tenotomy model receiving GANT58 administration, *n* = 12 for 1, 4, 7 weeks, *n* = 22 for 10 weeks), 4 SAG (a smoothened agonist) groups euthanized at 1, 4, 7, and 10 weeks separately (Achilles tenotomy model receiving SAG administration, *n* = 12 for 1, 4, 7 weeks, *n* = 22 for 10 weeks), 4 sham groups euthanized at 1, 4, 7, and 10 weeks separately (sham surgery, *n* = 15 for each group) (Figure 1A). The right Achilles tendons harvested at each time point were subjected to further analyses. The group allocation at the different stages of the experiment used blinding.

### 2.2. GANT58 and SAG Administration

For in vivo experiments, GANT58 (Abmole; M1812) dissolved in dimethyl sulfoxide (DMSO) was injected intraperitoneally (i.p.) into the mice every day (50 mg/kg, 100 μL/mouse) from day 1 to week 6 after Achilles tenotomy. SAG (Beyotime; SF6836) dissolved in DMSO was injected i.p. into the mice every day (10 mg/kg, 100 μL/mouse) from day 1 to week 6 postinjury. An equal volume of DMSO as the GANT58 or SAG solvent was administered to the vehicle groups. In vitro, cells were treated with gradient concentrations of GANT58 (0.25, 2.5, 25 mM) and SAG (0.02, 0.2, 2 mM) dissolved in DMSO separately. An equal amount of the vehicle was administered as a control (Figure 1B).

### 2.3. Microcomputed Tomography (Micro-CT)

The distal hind limbs harvested from the mice processed with GANT58 (*n* = 10), SAG (*n* = 10), or vehicle (*n* = 10) at 10 weeks after Achilles tenotomy were fixed in 4% paraformaldehyde (PFA) at 4 °C for at least 24 h and subjected to a micro-CT scanner (Skyscan 1176; Bruker, Billerica, MA, USA) at 50 kVp and 200 µA. Data were analysed at a threshold of 255 for the detection of ectopic mineralized bones.

### 2.4. Histologic, Histochemical and Immunohistochemical (IHC) Analyses

The distal hind limbs after harvest were fixed in 4% PFA for at least 24 h, decalcified with an ethylene diamine tetraacetic acid (EDTA) solution for 7–10 days, and paraffin-embedded. The Achilles tendons were made into 5-μm longitudinal sections and histologically stained with haematoxylin and eosin (H&E) or Alcian blue according to standard protocols. The calcified HO area in H&E staining and the positive area of Alcian blue staining at each slide on three contiguous sections per sample in each group were quantified on ImageJ. For IHC detection of Hh signalling-related molecules (e.g., SHH, IHH, SMO, GLI1), osteogenic biomarkers (e.g., osteocalcin (OCN) and runt-associated transcription factor 2 (RUNX2)) and chondrogenic biomarkers (e.g., aggrecan (AGG) and α-1 chain of type II collagen (COL2A1)), the samples were cultured with relevant primary antibodies following standard instructions. The antibodies included anti-SHH (20697-1-AP), anti-IHH (13388-1-AP), anti-GLI1 (66905-1-lg), anti-AGG (13880-1-AP) (all Proteintech), anti-SMO (DF5152), anti-OCN (DF12303) (both Affinity), anti-RUNX2 (ET1612-47), and anti-COL2A1 (ER1906-49) (both HUABIO). The positive cell ratio was quantified in three randomly selected fields using ImageJ.

### 2.5. Gene Expression Analysis

Total RNA of the samples from animals and cells was extracted by TRIzol and reverse-transcribed into complementary DNA (cDNA) by PrimeScript^TM^ RT Master Mix (both Takara, San Jose, CA, USA). Quantitative real-time polymerase chain reaction (qRT–PCR) was conducted using SYBR Premix Ex Taq^TM^ II (Takara) on an ABI QuantStudio5 (Applied Biosystems, Foster City, CA, USA). Data were standardized to glyceraldehyde-3-phosphate dehydrogenase. Relative expression of each gene was computed using 2^−ΔΔCt^. The primer sequences of relevant genes are shown in Supplementary Table S1.

### 2.6. Cells Culture

TDSCs were isolated from the Achilles tendons of 6- to 8-week-old SD female rats as described previously [25,26,27]. After the tendon sheath was stripped off, the tendon samples were cut into small pieces and digested with 3 mg/mL collagenase I (Sigma, C0130, Burlington, MA, USA) for 3 h at 37 °C. After the digestion solution containing TDSCs was passed through a 70 μm cell strainer, the released TDSCs were resuspended into single-cell suspensions and cultivated in plates with low-glucose Dulbecco’s modified Eagle medium (DMEM) added with 15% foetal bovine serum (FBS) (both Gibco, Vacaville, CA, USA) and 1% penicillin/streptomycin. Then cells were collected with 0.25% Trypsin-EDTA (Gibco) for passage. The third to fifth passage cells were chosen for in vitro experiments. The medium was changed every 3 days.

For osteogenic induction, 1.0 × 10^5^ cells/well in 6-well plates and 5.0 × 10^4^ cells/well in 12-well plates were grown for gene detection and alkaline phosphatase (ALP) staining, respectively. After reaching 80% confluence, the medium was changed to an osteogenic inducing medium composed of high-glucose DMEM (Gibco) containing 10^−8^ M dexamethasone, 50 μg/mL vitamin C, 10 mM β-glycerol phosphate (all Sigma–Aldrich, St. Louis, MO, USA), 10% FBS and 1% penicillin/streptomycin. The medium was renewed every 2 days. On day 7 of culture, the ALP activity of TDSCs seeded into 12-well plates was tested using ALP staining. On day 7 and 14, cells seeded into 6-well plates were used for RNA extraction.

To evaluate chondrogenesis in a micromass culture system [28], 2.0 × 10^5^ cells in a volume of 10 μL were carefully seeded into the centre of each well of a 24-well plate. After the cells adhered at 37 °C for 3–5 h, each well was added with a chondrogenic induction medium composed of high-glucose DMEM containing 1 mM sodium pyruvate (Gibco), 1% insulin-transferrin-selenium and 40 μg/mL proline (both Sigma–Aldrich, Burlington, MA, USA), 50 μg/mL vitamin C, 10^−7^ M dexamethasone, 10 ng/mL TGF-β3 (Peprotech, Westlake Village, CA, USA), and 1% penicillin/streptomycin. The medium was renewed every other day. On day 7, micromass cultures were used for RNA extraction and stained with Alcian blue.

### 2.7. ALP Staining

TDSCs seeded into 12-well plates were induced with an osteogenic medium using different concentrations of GANT58 and SAG for 7 days, fixed in 4% PFA for 30 min, and then stained with a BCIP/NBT ALP colour development kit (Beyotime, C3206) as instructed. The positive rates of ALP staining were quantified on ImageJ.

### 2.8. Alcian Blue Staining

TDSCs seeded in the micromass culture system were induced using a chondrogenic medium with different concentrations of GANT58 and SAG for 7 days, fixed in 4% PFA for 30 min, and then stained using an Alcian blue stain kit (Solarbio, G1563) at pH 1.0. The positive rates of Alcian blue staining were quantified using ImageJ.

### 2.9. Measurement of Intracellular ROS Levels

The levels of ROS were detected by a reactive oxygen species assay kit (BestBio, BB-4705) according to the manufacturer’s protocol. The results were evaluated by flow cytometer and fluorescent microscopy.

### 2.10. Statistical Methods

Data obtained from at least three independent experiments are shown as mean ± standard deviation (SD) of the mean. Inter-group differences were computed using two-tailed Student’s *t* test and one-way analysis of variance. The significance levels are * *p* < 0.05, ** *p* < 0.01, *** *p* < 0.001, and **** *p* < 0.0001.

## 3. Results

### 3.1. Hh Signalling Is Activated during the Progression of Heterotopic Ossification in the Tendon after Achilles Tenotomy

**Figure 2:**
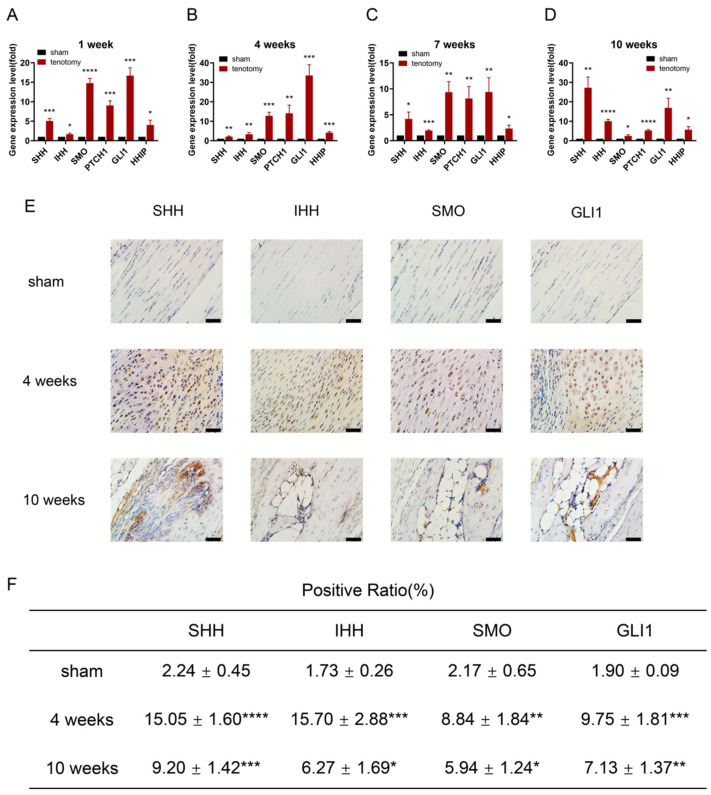
Hedgehog signalling is upregulated in tendons after Achilles tenotomy. (**A**–**D**) qRT–PCR analysis of the Hh signalling-related molecules SHH, IHH, SMO, PTCH1, GLI1, and HHIP in healthy and tenotomy groups at 1 (**A**), 4 (**B**), 7 (**C**), 10 (**D**) weeks. (**E**) Images of immunohistochemical staining of SHH, IHH, SMO, and GLI1 in the healthy and tenotomy groups at 4 and 10 weeks. Scale bar = 20 μm. (**F**) Quantification of immunohistochemical staining of SHH, IHH, SMO, and GLI1. * *p* < 0.05, ** *p* < 0.01, *** *p* < 0.001, **** *p* < 0.0001.

To determine whether Hh signalling is essential in the formation of trauma-induced tendon ossification, we collected the injured tendon specimens from mice at 1, 4, 7, and 10 weeks after Achilles tenotomy for further analyses. At the gene level, qRT–PCR implied the specific gene expressions of Hh signalling-related molecules (e.g., SHH, IHH, SMO, PTCH1, GLI1, HHIP) significantly increased at all time points (Figure 2A–D). At the protein level, IHC staining showed that SHH, IHH, SMO, and GLI1 were significantly upregulated during HO development in the injured tendons at 4 and 10 weeks, compared to the sham groups (Figure 2E,F). These results support that Hh signalling is distinctly upregulated during ossification formation in the Achilles tenotomy model, suggesting that Hh signalling is very likely involved in trauma-induced tendon HO.

### 3.2. Inhibition of Hh Signalling Restricts Posttraumatic Tendon Endochondral Ossification

**Figure 3:**
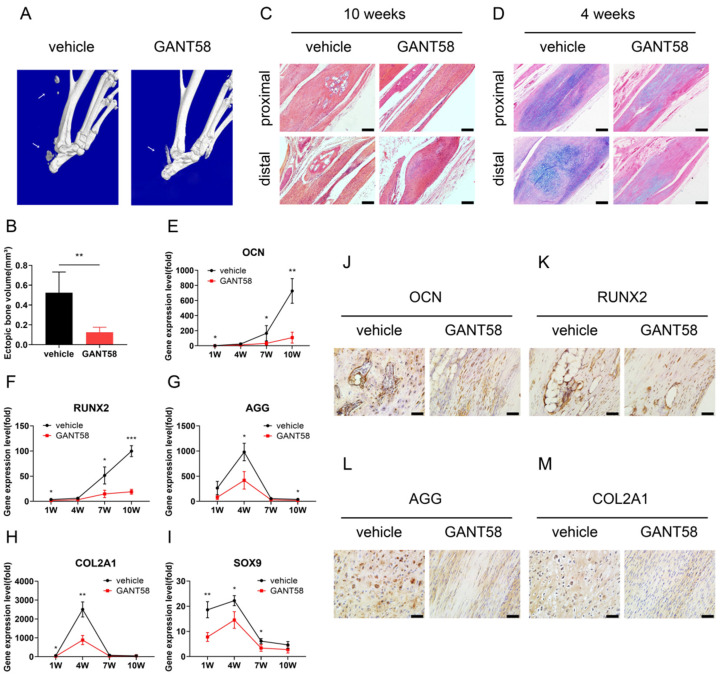
Inhibition of Hedgehog signalling suppresses formation of endochondral ossification in injured tendons. (**A**) Micro-CT images of the vehicle and GANT58 groups 10 weeks postinjury. (**B**) Quantification of the volume of ectopic mineralized bone. (**C**) H&E staining of the vehicle and GANT58 groups 10 weeks postinjury. Scale bar = 100 μm. (**D**) Alcian blue staining of the vehicle and GANT58 groups 4 weeks postinjury. Scale bar = 100 μm. (**E**–**I**) qRT–PCR analysis of osteogenic-specific genes such as OCN (**E**) and RUNX2 (**F**) and chondrogenic-specific genes such as AGG (**G**), COL2A1 (**H**), and SOX9 (**I**) in tendon specimens of the vehicle and GANT58 groups 1, 4, 7, and 10 weeks postinjury. (**J**,**K**) Images of IHC staining of OCN (**J**) and RUNX2 (**K**) in the tendons of the vehicle and GANT58 groups 10 weeks postinjury. Scale bar = 20 μm. (**L**,**M**) Images of IHC staining of AGG (**L**) and COL2A1 (**M**) in the tendons of the vehicle and GANT58 groups 4 weeks postinjury. Scale bar = 20 μm. * *p* < 0.05, ** *p* < 0.01, *** *p* < 0.001.

GANT58, a GLI1 antagonist, was used to treat the mice from day 1 to week 6 after Achilles tenotomy, and tendons were sampled at 1, 4, 7, and 10 weeks. At 10 weeks, micro-CT uncovered obvious mineralized bone formation at the proximal and distal ends of the Achilles tendons in the vehicle-treated group, while GANT58 strongly protected the injured tendons from forming ectopic mineralized bones (Figure 3A,B).

Histologically, the H&E staining demonstrated that GANT58 significantly reduced the areas of ectopic bones in injured tendon sections at 10 weeks (0.24 ± 0.09-fold above vehicle group; *p* < 0.01) (Figure 3C). Because endochondral ossification is a significant part of bone formation [6], we stained the tendon sections from the vehicle and GANT58 groups with Alcian blue at 4 weeks and detected positive cartilage nodules in both groups, indicating that tendon ossification occurred through endochondral ossification. More importantly, formation of cartilage nodules was heavily suppressed in the GANT58 group compared to the vehicle group (30.85 ± 3.06% vs. 8.15 ± 5.44%; *p* < 0.001) (Figure 3D).

Cartilage is generated and matures to bone during the development of endochondral ossification. In our specimens, the expressions of osteogenic- and chondrogenic-specific genes were confirmed by qRT–PCR at all time points during tendon ossification formation. Gene transcript values were normalized to healthy tendons. Results revealed that GANT58 significantly downregulated the expressions of bone-related genes such as OCN and RUNX2 at 7 and 10 weeks when mature ectopic mineralized bone was gradually formed (Figure 3E,F). Additionally, GANT58 restricted the expression of cartilagic genes (e.g., AGG, COL2A1, SOX9) at 4 weeks when cartilage tissues were formed and matured. Moreover, expression levels of chondrogenesis marker genes decreased during the osteogenic phase at 7 and 10 weeks (Figure 3G–I).

At 10 weeks, IHC showed that OCN and RUNX2 proteins were positively expressed in both the vehicle and GANT58 groups. However, OCN and RUNX2 staining was strongly detected in the newly-formed bone tissues of the vehicle group, but was only found in a few cells in the GANT58 group, suggesting GANT58 significantly reduced OCN (19.99 ± 1.60% vs. 6.92 ± 1.83%; *p* < 0.001) and RUNX2 (15.17 ± 1.55% vs. 7.21 ± 1.98%; *p* < 0.01) protein expressions (Figure 3J,K). In the chondrogenic stage at week 4, the chondrocytes in the new cartilage tissues of the vehicle group were markedly positive for AGG and COL2A1. In the GANT58 group, however, we observed few chondrocytes and weak staining of AGG (8.11 ± 1.19% vs. 1.84 ± 0.81%; *p* < 0.01) and COL2A1 (3.91 ± 0.40% vs. 1.44 ± 0.69%; *p* < 0.01) (Figure 3L,M).

### 3.3. Activation of Hh Signalling Promotes Posttraumatic Tendon Endochondral Ossification

**Figure 4:**
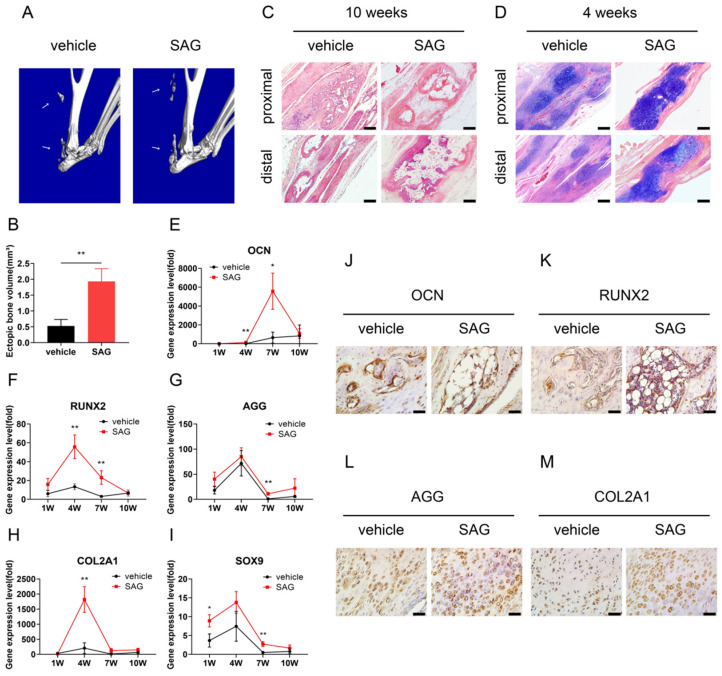
Upregulation of Hedgehog signalling promotes formation of endochondral ossification in injured tendons. (**A**) Micro-CT images of the vehicle and SAG groups 10 weeks postinjury. (**B**) Quantification of the volume of ectopic mineralized bone. (**C**) H&E staining of the vehicle and SAG groups 10 weeks postinjury. Scale bar = 100 μm. (**D**) Alcian blue staining of the vehicle and SAG groups 4 weeks postinjury. Scale bar = 100 μm. (**E**–**I**) qRT–PCR analysis of osteogenic-specific genes such as OCN (**E**) and RUNX2 (**F**) and chondrogenic-specific genes such as AGG (**G**), COL2A1 (**H**), and SOX9 (**I**) in tendon specimens of the vehicle and SAG groups 1, 4, 7, and 10 weeks postinjury. (**J**,**K**) Images of IHC staining of OCN (**J**) and RUNX2 (**K**) in the tendons of the vehicle and SAG groups 10 weeks postinjury. Scale bar = 20 μm. (**L**,**M**) Images of IHC staining of AGG (**L**) and COL2A1 (**M**) in the tendons of the vehicle and SAG groups 4 weeks postinjury. Scale bar = 20 μm. * *p* < 0.05, ** *p* < 0.01.

Next, we used SAG, a SMO agonist, to treat the mice after Achilles tenotomy. At 10 weeks, while micro-CT showed obvious ectopic bone generation in the Achilles tendons of the vehicle group, SAG more significantly accelerated the formation of ectopic bone tissues in the injured tendons (Figure 4A,B).

The Achilles tendons were histologically detected using H&E and Alcian Blue staining at 10 and 4 weeks, separately. Compared to the vehicle group, SAG significantly promoted not only ectopic bone formation in the injured tendons at 10 weeks (3.16 ± 0.48-fold above vehicle group; *p* < 0.05) (Figure 4C), but also cartilage nodule formation at 4 weeks (25.43 ± 4.90% vs. 33.78 ± 1.36%; *p* < 0.05) (Figure 4D), indicating Hh signalling upregulation heavily stimulates tendon HO development through endochondral ossification.

The qRT–PCR showed that SAG significantly upregulated the expressions of bone-related genes (e.g., OCN and RUNX2) at 4 and 7 weeks (Figure 4E,F) as well as cartilage-related genes such as AGG and SOX9 at 7 weeks and COL2A1 at 4 weeks (Figure 4G–I), indicating that SAG accelerates chondrogenesis and osteogenesis of injured tendons during endochondral ossification.

At 10 weeks, IHC revealed that OCN and RUNX2 proteins were detectable in the newly formed ectopic bone in both the vehicle and SAG groups. However, the SAG group exhibited stronger expression of OCN (14.21 ± 2.21% vs. 23.73 ± 5.23%; *p* < 0.05) and RUNX2 (13.30 ± 2.35% vs. 21.23 ± 3.28%; *p* < 0.05) proteins in osteocytes in more ossification tissues than the vehicle group (Figure 4J,K). At 4 weeks, the staining of AGG (11.56 ± 2.83% vs. 20.38 ± 1.31%; *p* < 0.01) and COL2A1 (9.92 ± 0.53% vs. 17.79 ± 3.57%; *p* < 0.05) in chondrocytes of cartilage tissues was positive in both groups, but was significantly more intense in the SAG group (Figure 4L,M).

### 3.4. Regulation of Hh Signalling Affects Osteogenesis and Chondrogenesis of TDSCs In Vitro

The TDSCs isolated from SD rats were incubated and used to detect the impacts of GANT58 and SAG on osteogenic and chondrogenic differentiation potentials.

**Figure 5:**
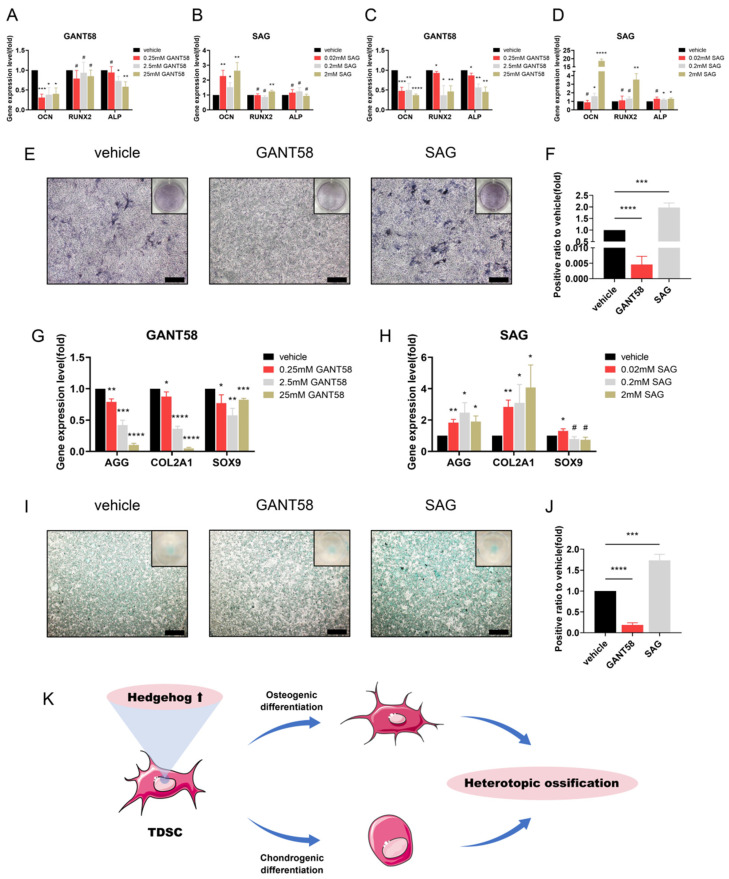
Hedgehog signalling regulates osteogenic and chondrogenic differentiation in tendon-derived stem cells (TDSCs). (**A**) qRT–PCR analysis of OCN, RUNX2, and alkaline phosphatase (ALP) in TDSCs treated with GANT58 at different concentrations under weeklong osteogenic induction. (**B**) qRT–PCR analysis of OCN, RUNX2, and ALP in TDSCs treated with SAG at different concentrations under weeklong osteogenic induction. (**C**) qRT–PCR analysis of OCN, RUNX2, and ALP in TDSCs treated with GANT58 at different concentrations under two weeks of osteogenic induction. (**D**) qRT–PCR analysis of OCN, RUNX2, and ALP in TDSCs treated with SAG at different concentrations under two weeks of osteogenic induction. (**E**) ALP staining of TDSCs treated with 25 mM GANT58 and 2 mM SAG under weeklong osteogenic induction. Scale bar = 200 μm. (**F**) Quantification of ALP staining. (**G**) qRT–PCR analysis of AGG, COL2A1, and SOX9 in TDSCs treated with GANT58 at different concentrations under weeklong chondrogenic induction. (**H**) qRT–PCR analy Scheme 2. A1 and SOX9 in TDSCs treated with SAG at different concentrations under weeklong chondrogenic induction. (**I**) Alcian blue staining of TDSCs treated with 25 mM GANT58 and 2 mM SAG under weeklong chondrogenic induction. Scale bar = 200 μm. (**J**) Quantification of Alcian blue staining. (**K**) Schematic figure showing that Hh signalling affects osteogenic and chondrogenic differentiation in TDSCs, which are involved in tendon heterotopic ossification. * *p* < 0.05, ** *p* < 0.01, *** *p* < 0.001, **** *p* < 0.0001, # no significance.

As for the effect on osteogenic differentiation, TDSCs were processed with GANT58 and SAG under osteogenic induction. After 7 and 14 days, the gene expressions of osteogenic markers (e.g., OCN, RUNX2, ALP) were suppressed after treatment with GANT58, but promoted after treatment with SAG (Figure 5A–D). After 7 days, GANT58 reduced ALP staining, indicating osteogenic differentiation was suppressed with downregulation of Hh signalling, while SAG increased ALP staining, suggesting osteogenic differentiation was promoted with upregulation of Hh signalling (Figure 5E,F).

A micromass culture system was used for chondrogenic induction. After 7 days of culture, qRT–PCR demonstrated gene expressions of chondrogenic markers, including AGG, SOX9, and COL2A1, were suppressed in TDSCs in response to GANT58 treatment, but were promoted in response to SAG treatment (Figure 5G,H). At the same time, the GANT58 group exhibited less intense Alcian blue staining, suggesting chondrogenic differentiation was suppressed with downregulation of Hh signalling, while the SAG group displayed more intense staining, indicating chondrogenic differentiation was promoted with upregulation of Hh signalling (Figure 5I,J).

In summary, osteogenesis and chondrogenesis during endochondral ossification of tendons are greatly regulated by Hh signalling at the cellular level. Thus, TDSCs are likely to be one of the targets for GANT58 and SAG and are in charge of tendon ossification involving Hh signalling in this model.

### 3.5. Hh Signalling Regulates Antioxidant Pathway in Osteogenic Differentiation of TDSCs

**Figure 6:**
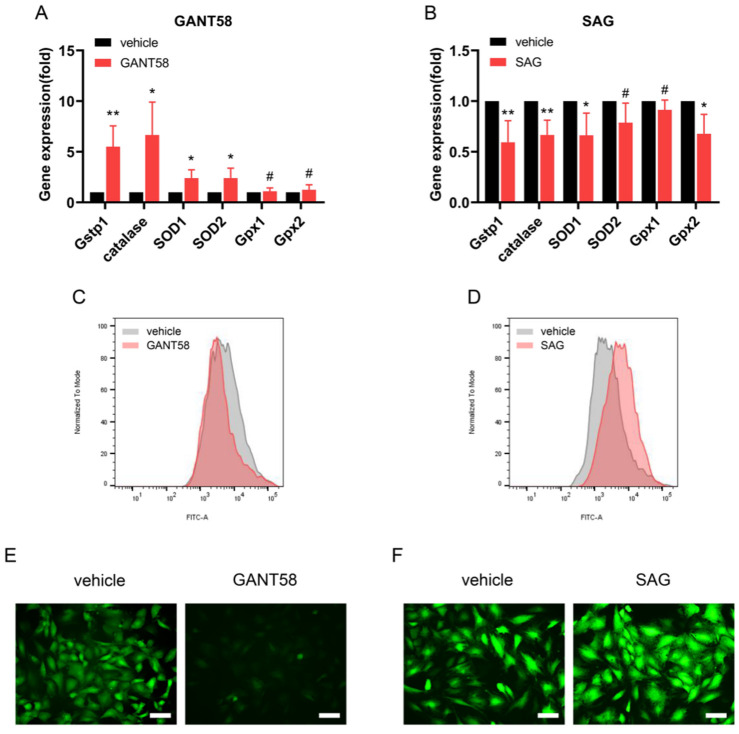
Hedgehog signalling regulates antioxidant pathway in osteogenic differentiation of TDSCs. (**A**) qRT–PCR analysis of antioxidant related genes in TDSCs treated with 25 mM GANT58 under weeklong osteogenic induction. (**B**) qRT–PCR analysis of antioxidant related genes in TDSCs treated with 2 mM SAG under weeklong osteogenic induction. (**C**,**E**) TDSCs were treated with 25 mM GANT58 under osteogenic induction for 48 h, followed by detection of reactive oxygen species (ROS) levels by flow cytometry (**C**) and fluorescent microscopy (**E**). (**D**,**F**) TDSCs were treated with 2 mM SAG under osteogenic induction for 48 h, followed by detection of ROS levels by flow cytometry (**D**) and fluorescent microscopy (**F**). * *p* < 0.05, ** *p* < 0.01, # no significance.

Since ROS has been reported to be involved in the formation of tendon HO [22], we next examined whether Hh signalling regulates the antioxidant pathway and ROS generation during osteogenic differentiation of TDSCs. After weeklong osteogenic induction, most of the gene expressions of antioxidant related markers (e.g., glutathione S-transferase pi 1 (Gstp1), catalase, superoxide dismutase 1 (SOD1), superoxide dismutase 2 (SOD2), glutathione peroxidase 1 (Gpx1), glutathione peroxidase 2 (Gpx2)) of TDSCs were promoted after treatment with GANT58, but suppressed after treatment with SAG (Figure 6A,B). Flow cytometry and fluorescent microscopy images showed that GANT58 reduced the levels of ROS of TDSCs under osteogenic induction for 48 h (0.84 ± 0.04-fold above vehicle group in flow cytometry; *p* < 0.01) (19.77 ± 4.03 AU vs. 6.43 ± 0.91 AU in mean fluorescence intensity; *p* < 0.0001), while SAG promoted ROS generation under the same conditions (1.47 ± 0.21-fold above vehicle group in flow cytometry; *p* < 0.05) (19.43 ± 2.79 AU vs. 34.53 ± 6.88 AU in mean fluorescence intensity; *p* < 0.01) (Figure 6C–F). These data suggest Hh signalling can regulate antioxidant pathway and affect ROS generation in osteogenic differentiation of TDSCs, and the antioxidant effect of Hh signalling may be partially involved in the regulation of tendon HO.

## 4. Discussion

Tendon ossification, one of the most common and serious complications after tendon injury, is detrimental to the normal repair of tendons and seriously affects tendon function. Therefore, it is of both basic scientific value and clinical significance to clarify the intervention targets and regulatory mechanisms of trauma-induced tendon ossification and explore effective drugs or new methods for targeting post-trauma tendon ossification.

Hh signalling is a highly conserved signal transducing pathway that is critically involved in embryonic growth and maturation, maintenance of the stable internal environment of organs, postinjury tissue repair and regeneration, and tumour occurrence, development, differentiation, and invasion [29]. Dysregulation of Hh signalling can disrupt musculoskeletal tissue homeostasis [30]. At present, research about the function of Hh signalling in tendon development, homeostasis maintenance, or postinjury tendon repair and HO is rare. Reportedly, Hh signalling is upregulated in tendon sheath tissues after tendon injury and promotes Bglap^+^ cell migration into the tendons, but the origin of Bglap^+^ cells or their specific role in repair was not clarified [31]. Additionally, spontaneous tendon ossification depends on the upregulation of Hh signalling in the Achilles tendons of MKX^−/−^ mice, but the role of Hh signalling in tendon ossification of healthy mice is unknown [32]. Moreover, intensified Hh signalling pathologically drives ectopic cartilage and bone generation in soft tissues [33] via heterotopic chondrogenesis and HO [34]. In animal models, gene-modulated ectopic Hh signalling is enough to initiate HO, while pharmacologic or genetic restriction of Hh signalling heavily weakens the severity of this condition [3]. Likewise, synovial chondromatosis, an induction of synovial tissue ossification, is linked with intensified canonical Hh signalling [35]. In contrast to cartilage and bone gain, higher Hh signalling is also related to cartilage deterioration and loss [36,37]. Thus, suitable Hh signalling normally participates in the ectopic inhibition and the maintenance and genesis of normtopic bone/cartilage. Notably, IHH, which guides Hh signalling within the developing limbs at birth, is expressed in an area of postmitotic, prehypertrophic chondrocytes that are just near the zone of proliferating chondrocytes [38,39,40] and is pivotal in endochondral ossification, and initiates osteoblast differentiation in the perichondrium [41]. However, the effect of IHH on endochondral ossification in tendons has not been investigated. Recently, a self-amplifying and self-propagating loop of YAP and SHH is reportedly a common mechanism of ectopic bone formation and expansion in mouse models of genetic HO and injury-induced HO. Upregulation of YAP and SHH are also found in samples from patients with HO [42]. These findings indicate that SHH may drive the process of formation of HO.

As reported extensively, Hh signalling directly acts on mesenchymal stem cells (MSCs) and regulates the direction of lineage differentiation [43,44,45]. A relatively clear conclusion holds that Hh signalling promotes the osteogenic and chondrogenic differentiation of MSCs [44] and inhibits adipogenic differentiation [44]. Current evidence supports that TDSC motion is necessary for tendon injury curing. During tendon restoration and reproduction, TDSCs can move to the injury site, differentiate into tenocytes, and substitute the abnormal tenocytes participating in the pathophysiological process [46], but due to postinjury changes in the local microenvironment, local stem cells can mis-differentiate into bones and cartilage [47], which is one of the reasons for the occurrence and development of tendon ossification. The pathological procedure of chronic tendinopathy generates some dysfunctional matrix parts (e.g., hypervascularity, acquisition of chondrocyte phenotypes, calcium formation). Erroneous differentiation of TDSCs is indicated to cause TDSC pool consumption and ectopic chondro-ossification [10]. However, for tendon tissues, it is unclear whether Hh signalling directly acts on TDSCs and regulates the postinjury differentiation direction of lineages. Our results provide evidence that Hh signalling may directly modulate the osteogenic and chondrogenic differentiation of TDSCs during the pathogenesis of tendon endochondral ossification. The maintenance of normal physiological function requires a balance between oxidation and antioxidation. Antioxidant systems such as Gstp1, catalase, SOD, and Gpx sever the purpose of elimination of excessive ROS [22]. When overwhelming ROS break the balance between oxidative stress and antioxidant systems, oxidative stress contributes to pathophysiological processes [48]. For MSCs, upregulation of ROS impairs osteogenic differentiation and stimulates chondrogenesis [49]. For tendon stem/progenitor cells, high level of ROS promote osteogenesis and chondrogenesis differentiation and participate in the pathogenesis of tendon HO [22]. These results imply that ROS may have different effects on osteogenesis and chondrogenesis in different cells and disease models. Hh signalling has been reported to regulate osteogenesis in MSCs through modulation of ROS [50]. Our study finds that Hh signalling regulates antioxidant pathway and affects ROS generation in osteogenic differentiation of TDSCs, suggesting the antioxidant effect of Hh signalling may be partially involved in the regulation of tendon HO.

Tissue injury can cause local inflammation and macrophage gathering, which further result in an assembly of osteogenic factors, including BMPs. This probably disrupts local stem/progenitor cells and drives them to follow osteogenic routes with HO development, implying that such disruption can be a major cellular mechanism of HO [7]. Kan et al. demonstrate that niche-dwelling progenitor/stem cells and niche supportive cells (including mast cells, neurites, vasculature, and macrophages) constitute injury-induced local microenvironment (MSC niche) of HO. Through feedback and non-cell autonomous mechanisms, BMP and Hh signalling co-regulate the formation of the MSC niche, which may initiate the pathological osteogenic cascade [51]. BMS-833923, a SMO antagonist, has been reportedly shown to remarkably inhibit osteoblast differentiation of human skeletal (mesenchymal) stem cells (hMSCs). Global gene expression profiling of BMS-833923-treated identifies many genes with significant changes, which markedly impact multiple signalling including inflammatory response signalling [43]. These studies suggest that our treatments may also have effects on immune cells such as monocytes and macrophages in the inflammatory stage. Other cells such as immune or vascular cells may also contribute to Hh family ligands. Further research is needed to determine whether formation of tendon HO in the Achilles tenotomy model is driven by a cell-autonomous Hh signalling activation of TDSCs or by factors from other cells.

GANT58, a small molecule inhibitor of GLI1, prevents GLI1-dependent transcription through the suppression of its post-translational modification [52]. As an Hh inhibitor, it shows therapeutic potential for age-related bone loss and tumour-induced bone disease [52,53]. SAG, a specific Hh agonist, has been shown to promote osteogenic differentiation and bone regeneration [54,55,56]. In this study, we consider the Hh as a whole signalling pathway and show that Hh is responsible for ectopic mineralized bone formation in injured tendons. The use of GANT58 to downregulate Hh signalling suppresses, while SAG as an upregulator promotes, osteogenesis and chondrogenesis during the formation of trauma-induced tendon ossification both in vivo and in vitro. However, there are some limitations to our findings: (1) This study does not further explore the regulatory mechanism of Hh signalling-related molecules during the trauma-induced tendon ossification, which is another interesting issue that deserves further investigation. (2) We do not go further to test whether there is any side effect on the musculoskeletal system after drug treatment. The development of more precisely targeted drugs requires further exploration. (3) All experiments are limited to animals and cells, which requires that the effect of GANT58 on human tendons shall be investigated in clinical trials. All the same, we confirm that Hh signalling can regulate the formation of trauma-induced tendon ossification. This study offers direct proof that Hh signalling may be a potential clinical therapeutic target for trauma-induced tendon ossification.

## 5. Conclusions

In conclusion, Hh signalling is activated in post-trauma tendon ossification and the regulation of Hh signalling affects the formation of tendon ossification by modulating the differentiation direction of TDSCs. Hh signalling can regulate antioxidant pathway and affect ROS generation in osteogenic differentiation of TDSCs. Thus, we demonstrate that a classic signalling pathway directly and remarkably regulates ossification formation in injured tendons, which may be an extremely meaningful functional study. Both in vivo and in vitro results suggest that GANT58 inhibits trauma-induced tendon ossification by restricting chondrogenesis and osteogenesis of tendons. Therefore, targeting Hh signalling by GANT58 may be a potential therapeutic strategy for useful prevention of trauma-induced tendon ossification.

## Data Availability

Data is contained within the article.

## Supplementary Materials

The following supporting information can be downloaded at: https://www.mdpi.com/article/10.3390/antiox11112265/s1, Supplementary Table S1 showing the primer sequences for quantitative real-time polymerase chain reaction analysis of gene expression conducted in this study.
