# Supplementary material for: Hedgehog Signalling Contributes to Trauma-Induced Tendon Heterotopic Ossification and Regulates Osteogenesis through Antioxidant Pathway in Tendon-Derived Stem Cells
Source: Antioxidants (Basel). 2022 Nov 16;11(11):2265. doi: 10.3390/antiox11112265 (PMC9686894; doi:10.3390/antiox11112265)
Supplement: Supplementary file 1 [file antioxidants-11-02265-s001.zip › antioxidants-1908281-supplementary.pdf]

Supplementary Table S1 Primer sequences used in qRT–PCR

Primer name	Forward sequence	Reverse sequence
Mouse GAPDH	AGGTCGGTGTGAACGGATTTG	TGTAGACCATGTAGTTGAGGTCA
Mouse SHH	GATGAGGAAAACACGGGAGC	CTGCTCGACCCTCATAGTGT
Mouse IHH	CGTGCATTGCTCTGTCAA GT	CTCGATGACCT GGAAAGCTC
Mouse SMO	GGACATGCACAGCTACATCG	CTCGGCAAACAATCTCTCGG
Mouse PTCH1	GCCTTCGCTGTGGGATTAAAG	CTTCTCCTATCTTCTGACGGGT
Mouse GLI1	TCTGTGATGGGCAATGGTCT	TCTGGGGTGGGATCAGGATA
Mouse HHIP	CCACTGACCTCCGATTGCTC	TGCAGCAGCACTTGCCAG
Mouse OCN	GTCCCTCACCCTCCCAAAAG	CAGGAGGGCAATAAGGTAG
Mouse RUNX2	GGCCAGAGGCAGAAGTCA	CAAGTAGCCAGGTTCAACGA
Mouse AGG	CGTTGCAGACCAGGAGCAAT	CTCGGTCATGAAAGTGGCG
Mouse Collagen II	GGTGAGCCATGATCCGCC	TGGCCCTAATTTTCGGGCATC
Mouse SOX9	GTGCAAGCTGGCAAAGTTGA	TGCTCAGTTCACCGATGTCC
Rat GAPDH	GGCATTGCTCTCAATGACAA	TGTGAGGGAGATGCTCAGTC
Rat OCN	CGCGTAAACGCCCTTTTGAT	AGTCTTGCAGCACCCGTAAA
Rat RUNX2	GTCGTCAGACCGAGAAGTGG	TCAAGTTCGAGGAAGCCGTG

Rat ALP	GCCTTACCAACTCATTTGTGC	CATACCATCTCCCAGGAACATG
Rat AGG	CAGTGCGATGCAGGCTGGCT	CCTCCGGCACTCGTTGGCTG
Rat Collagen II	CTCATCCAGGGCTCCAATGAT	TCTGTGATCGGTACTCGATGA
Rat SOX9	AGTACCCGCATCTGCACAAC	ACTTGTAATCGGGGTGGTCT
Rat Gstp1	CAGCTATGCCACCGTACACC	AGCTGCCCATACAGACAAGTG
Rat catalase	AGGGATGCCATGTTGTTTCC	AAGGTGTGTGAGCCATAGCC
Rat SOD1	CCGGGGAAGCATGGCG	GCAGTGGTACAGCCTTGTG
Rat SOD2	CGCGACCTACGTGAACAATC	CTCCAGCAACTCTCCTTTGG
Rat Gpx1	CAGTTCGGACATCAGGAGAATGG	GGAAGGTAAAGAGCGGGTGAG
Rat Gpx2	AGTTCGGACATCAGGAGAACTG	GGTCGTCATAAGGGTAGGGC
